# Genetic Diversity and Population Structure of Chinese Foxtail Millet [*Setaria italica* (L.) Beauv.] Landraces

**DOI:** 10.1534/g3.112.002907

**Published:** 2012-07-01

**Authors:** Chunfang Wang, Guanqing Jia, Hui Zhi, Zhengang Niu, Yang Chai, Wei Li, Yongfang Wang, Haiquan Li, Ping Lu, Baohua Zhao, Xianmin Diao

**Affiliations:** * National Key Facility for Crop Gene Resources and Genetic Improvement (NFCRI), Institute of Crop Science, Chinese Academy of Agricultural Sciences (CAAS), Beijing 100081, China; § Institute of Millet Crops, Hebei Academy of Agricultural and Forestry Science, Shijiazhuang 050031, China; ‡ College of Life Sciences, Hebei Normal University, Shijiazhuang 050012, China, and; § Department of Biology and Chemistry, Hebei Teachers’ College for Nationality, Chengde 067000, China

**Keywords:** foxtail millet, landrace, population structure, linkage disequilibrium

## Abstract

As an ancient cereal of great importance for dryland agriculture even today, foxtail millet (*Setaria italica*) is fast becoming a new plant genomic model crop. A genotypic analysis of 250 foxtail millet landraces, which represent 1% of foxtail millet germplasm kept in the Chinese National Gene Bank (CNGB), was conducted with 77 SSRs covering the foxtail millet genome. A high degree of molecular diversity among the landraces was found, with an average of 20.9 alleles per locus detected. STRUCTURE, neighbor-jointing, and principal components analyses classify the accessions into three clusters (topmost hierarchy) and, ultimately, four conservative subgroups (substructuring within the topmost clusters) in total, which are in good accordance with eco-geographical distribution in China. The highest subpopulation diversity was identified in the accessions of Pop3 from the middle regions of the Yellow River, followed by accessions in Pop1 from the downstream regions of the Yellow River, suggesting that foxtail millet was domesticated in the Yellow River drainage area first and then spread to other parts of the country. Linkage disequilibrium (LD) decay of less than 20 cM of genetic distance in the foxtail millet landrace genome was observed, which suggests that it could be possible to achieve resolution down to the 20 cM level for association mapping.

Foxtail millet [*Setaria italica* (L.) P. Beauv.] is one of the cereals in the Panicoideae tribe, distributed widely around warm and temperate regions in Asia, Europe, North America, Australia, and North Africa, used for grain or forage (Austin 2006). On the basis of recent archeological discoveries, it has been widely accepted that foxtail millet has been a very important cereal since ancient times in Eurasia and has greatly contributed to human civilizations both in Asia and Europe (Li and Wu 1996; Hunt *et al.* 2008; Barton *et al.* 2009). China has been identified as the center of origin of this special crop (Vavilov 1926), where foxtail millet was domesticated and selected as grain food as early as 8700 years ago (Lu *et al.* 2009). Precise figures on the amount of foxtail millet grown worldwide are not available, as foxtail millet production data are generally grouped with those of other small cereals, such as pearl millet (*Pennisetum glaucum*), finger millet (*Eleusine coracana*), and common millet (*Panicum miliaceum*). Taken together, these millet crops produced nearly 30 million tons (Mt) of grain in 2003 (FAO stat data 2004, http://faostat.fao.org/). It is reported that the annual growing area of foxtail millet in China is about 2 million ha with an annual total grain yield of about 6 Mt (Diao 2011). The growing area of foxtail millet in central Europe was about 15,000 ha in 2003 (FAO stat data 2004, http://faostat.fao.org/). However, foxtail millet breeding in China has made great progress, and its yield potential has been improved greatly in recent years (Diao 2011). Foxtail millet is an underutilized, drought-tolerant crop that stands to become much more important in a potentially much warmer and dryer future environment (Dekker 2003; Diao 2011; Dwivedi *et al.* 2011). Because of its small diploid genome (1C genome size = 515 Mb) and inbreeding nature, foxtail millet is a more tractable experimental model for grass functional genomics to investigate plant architecture, genome evolution, drought tolerance, and physiology in the bioenergy grasses (Doust *et al.* 2009). Green foxtail [*Setaria viridis* (L.) P. Beauv.], the wild ancestor of foxtail millet, is being used as a model species to investigate C_4_ photosynthesis (Brutnell *et al.* 2010). Following the release of the draft genome sequence of the Chinese cultivar Yugu 1, foxtail millet is on a fast track to becoming a plant genomic model crop (Doust *et al.* 2009; Minorsky 2009).

A diversified germplasm collection plays a key role in both breeding and genomic research for any crop species. The biggest collection of foxtail millet landraces is kept at the Chinese National Gene Bank (CNGB), Institute of Crop Science, Chinese Academy of Agricultural Sciences, Beijing, China, which has 26,670 accessions. Other collections are relatively small, including the International Crops Research Institute for the Semi-Arid Tropics (ICRISAT), Patancheru, India (1534 accessions from 26 countries), the National Institute of Agrobiological Sciences (NIAS), Tsukuba, Japan (1279 accessions), and the Plant Genetic Resources Conservation Unit (PGRCU), U.S. Department of Agriculture (766 accessions) (Doust *et al.* 2009).

Estimation of population diversity and structure of germplasm accessions could provide pivotal information for resource management, association mapping, and crop breeding. Genetic structure analysis of landraces have been carried out in many kinds of crop species, including rice (*Oryza sativa*) (Zhang *et al.* 2009), maize (*Zea mays*) (Liu *et al.* 2003), sorghum (*Sorghum bicolor*) (Barro-Kondombo *et al.* 2010), and soybean (*Glycine max*) (Li *et al.* 2008), which have contributed greatly to genomic analysis and breeding of these crops. Association mapping is an effective approach to detect QTL if information of population structure and linkage disequilibrium (LD) is available. Using SSR markers, association mapping has been successfully developed in rice (Jin *et al.* 2010), wheat (*Triticum aestivum*) (Kruger *et al.* 2004; Maccaferri *et al.* 2005), and barley (*Hordeum vulgare*) (Mather *et al.* 2004). Phenotypic analysis on morphological characteristics of foxtail millet landraces has demonstrated that they are highly diverse (Li and Wu 1996; Reddy *et al.* 2006). The genetic diversity of foxtail millet was assayed by the isoenzyme system (Jusuf and Pernes 1985) and RAPD markers (Schontz and Rether 1999). Jia *et al.* (2009) have characterized SSR diversity of 40 foxtail millet landraces collected from China. Van *et al.* (2008) detected the extent of LD in *Wx* gene sequences from a set of a worldwide collection of foxtail millet, and Wang *et al.* (2010) analyzed LD extent in nine selected genomic sequence fragments from 50 landrace accessions, with both studies revealing low levels of LD in foxtail millet. An updated report of population structure analysis by transposon display classified foxtail millet landraces into eight clusters that are closely related with geographical origins and suggest a monophyletic origin of foxtail millet domestication (Hirano *et al.* 2011). However, no detailed genomic SSR-based population structure study has been carried out on a large set of foxtail millet landraces, and we still know little about foxtail millet germplasm diversity and genome-wide extent of LD.

In this study, 250 accessions of foxtail millet landraces collected from China, which represent 1% of CNGB foxtail millet germplasm covering all geographical regions of China, were used in genetic diversity analysis using SSR markers. Population structure was inferred by software simulation, and linkage disequilibrium between pairs of SSR markers on a genome-wide scale was also analyzed. Result of this research will benefit germplasm conservation, association mapping, and genomic research in China and other countries, as well as identify suitable landraces for use in breeding.

## Material and Methods

### Landrace sampling

The majority of the foxtail millet landraces kept in CNGB was collected in the 1960s to 1980s, before pedigree selection based on hybridization for cultivar development was carried out. Those samples were collected from each eco-region of foxtail millet production located for the most part in Northern China. Based on the geographical distribution of these accessions, 250 landraces from different regions of China were randomly selected, which represent 1% of Chinese foxtail millet germplasm. Proportions of the number of accessions stored in CNGB from different geographical regions were considered as factors for sample selection. Therefore, the number of accessions from Northern China, where foxtail millet has a large growing area, was much bigger than that from Southern China, where foxtail millet is a relatively small crop. The purpose of the sampling strategy was to assemble a good representative set of accessions of foxtail millet landraces from all eco-geographical regions of China. The number of accessions from each province used in this study is listed in Table 1.

**Table 1:** Origin of landraces selected in this trial

Eco-regions	Province	No. of Accessions
Early-spring sowing region (ESR)	Heilongjiang	34
Spring sowing region (SR)	Shanxi	19
	Shannxi	14
	Gansu	16
	Inner Mongolia	17
	North Hebei	3
	Qinghai	10
	Tibet	5
	Xinjiang	9
	Ningxia	8
Summer and spring sowing region (SSSR)	Beijing	5
	South Hebei	19
	Henan	16
	Shandong	15
	Tianjin	2
	Jilin	11
	Liaoning	9
Southern China region (SCR)	Sichuan	8
	Hubei	4
	Hunan	3
	Guangxi	5
	Guangdong	1
	Jiangsu	5
	Zhejiang	1
	Jiangxi	5
	Hainan	4
	Guizhou	1
	Yunnan	1

### Genotyping by SSR markers

Template DNA was extracted by the CTAB method (Doyle 1991) from fresh leaves of sampled individuals (single individual per accession). Seventy-seven SSR markers were selected for analysis, covering the nine chromosomes of foxtail millet genome. The development of those SSR markers and their chromosome locations are listed in Table 2. All SSRs were labeled with different colors of fluorescent dye at the 5′ end of forward primer for PCR amplification (Applied Biosystems, USA). The PCR reaction mixture consisted of 1× Taq reaction buffer (Takala, with Mg^2+^), nucleotide dATP, dGTP, dCTP, dTTP (125 µM each), 0.1 µM primer, 1 unit of Taq DNA polymerase, and 20 ng template DNA. Fragment length of amplified DNA was measured using an ABI 3730XL analyzer. Polymorphism data were analyzed in GeneMapper (version 4.0). Microchecker version 2.2.3 (Oosterhout *et al.* 2004) was used for checking mistakes due to potential primer stuttering to make sure genotyping data are reliable.

**Table 2:** Chromosomal locations of SSR markers used for this study

Chromosome No.	No. of Markers	Marker Name
1	10	b165 p3 p8 p88 p33 b153 p58 b218 b126 p16
2	3	b242 p80 MPGD13*^a^*
3	8	b163 b186 p61 b225 p98 b101 MPGD32*^a^* MPGD44*^a^*
4	6	b109 p2 p42 b236 b247 p100
5	5	p17x b223 b237 si017*^b^* MPGA31*^a^*
6	4	p10 b263 b159 MPGA50*^a^*
7	6	p59 b123 b180 p45 p29 b200
8	3	b185 b258 si227*^b^*
9	15	b269 p4 p44 b241 b246 b187 b217 b166 b174 b171 p20 p41 b102 si119*^b^* si132*^b^*
Unclear	17	b224 b169 b266 b250 b249 b189 b182 b181 b117 p89 p78 b145 p32 p14 B1(AGDZ23-35)*^b^* X4*^c^* X298*^c^*

Primers from Jia *et al.* (2009), except as noted.

a Primers developed by Gupta *et al.* (2012).

b Primers developed by X. Diao’s lab, China (Table S2).

c Primer sources from BAC sequencing by Bennetzen’s lab, Georgia (Table S2).

### Genetic diversity and population structure

All summary statistics, such as allele number per locus, effective allele number per locus, Shannon’s information index, genotype number per locus, gene diversity, PIC values, observed homozygosity, genetic distance, and *Fst* test, were determined using POPGENE version 1.32 and PowerMarker version 3.25 (Liu and Muse 2005). Nei’s genetic distance (1983) was calculated and used for unrooted phylogeny reconstruction based on neighbor-joining methods as implemented by PowerMarker software, and the tree was visualized using MEGA 4.0 (Tamura *et al.* 2007). Principal component analysis (PCA) was carried out in GenALEX version 6.4 (Peakall and Smouse 2006). Analysis of molecular variance (AMOVA) was calculated by PowerMarker.

The model-based software program STRUCTURE version 2.3 (Pritchard *et al.* 2000) was used to infer population structure by a Bayesian approach using our SSR marker dataset. We deduced the optimal value of K (the number of clusters) by evaluating K = 1∼15. Admixture and non-admixture were allowed separately, and allele frequencies were assumed to be correlated or independent in these two models, respectively. Length of burn-in of the Markov Chain Monte Carlo (MCMC) iterations was set to 100,000, and data were collected over 100,000 MCMC iterations in each run. Twenty iterations per K were conducted. The optimal value of K was identified using both the ad hoc procedure introduced by Pritchard *et al.* (2000) and the method developed by Evanno *et al.* (2005). Genetic diversity, private allele number (Table 3), and divergence (Table 4) estimates were calculated for the different clusters identified by structure analysis. Substructure within each main cluster was detected by the same approach mentioned above using STRUCTURE version 2.3.

**Table 3:** Molecular diversity of model-based subpopulations inferred by STRUCTURE

Subpopulation	Sample Size	Genotype No./Locus	Allele No./Locus	Gene Diversity/Locus	PIC/Locus	Population-specific Allele No.
Pop1	96	15.96	14.54	0.81	0.79	153
Pop2N	33	9.57	9.10	0.75	0.72	43
Pop2S	24	9.66	9.50	0.78	0.76	79
Pop3	97	16.94	15.88	0.81	0.79	239

PIC, Polymorphism information content.

**Table 4:** Pairwise estimates of *Fst* and Nei’s genetic distance based on 77 SSR loci among four model based subpopulations

Subpopulation	Pop1	Pop2N	Pop2S	Pop3
Pop1		2.1345	2.3403	2.7991
Pop2N	0.3117		2.4809	2.1574
Pop2S	0.3384	0.4003		2.3038
Pop3	0.2095	0.3241	0.3498	

*Fst* estimates appear above the diagonal and pairwise genetic distance appears below the diagonal.

### Linkage disequilibrium

Linkage disequilibrium was evaluated for each pair of SSR loci by TASSEL, both on all landraces individually and on the clusters as inferred by STRUCTURE. D’ and r^2^ LD measures modified for loci were used (Hedrick 1987; Weir 1996). Significance (*P* value) of D’ for each SSR pair was determined by 100,000 permutations.

## Results

### Overall genetic diversity

All 77 markers were polymorphic across the 250 accessions, and 1612 alleles were detected (supporting information, Table S1). The average number of alleles per locus was 20.9351, ranging from 6 to 47. The average number of effective alleles per locus was 9.9101, ranging from 2.6732 to 23.8019. The average number of Shannon’s information index was 2.3868, ranging from 1.3196 to 3.3637. For genotype, the average number per locus was 24.5455, ranging from 6 to 46. As for gene diversity, the average number for each locus was 0.8571, ranging from 0.6310 to 0.9580. PIC value for markers was 0.8420 on average, ranging from 0.5937 to 0.9563. Homozygosity per locus on average was 0.9859, ranging from 0.9440 to 1 (Table 5). Average homozygosity was close to 100%, so indeed foxtail millet accessions are very much close to inbred lines.

**Table 5:** Genetic diversity of 250 foxtail millet landrace accessions assessed by 77 SSR markers

Per Locus	Na	Ne	I	Genotype No.	Gene Diversity	PIC	Obs_Hom
Average	20.9351	9.9101	2.3868	24.5455	0.8571	0.8420	0.9859
Range	6∼47	2.6732∼23.8019	1.3196∼3.3637	6∼46	0.6310∼0.9580	0.5937∼0.9563	0.9440 ∼1
SD	8.9873	5.4482	0.5806	10.0322	0.0906	0.1041	0.0126

Na, observed number of alleles; Ne, effective number of alleles (Kimura and Crow 1964); I, Shannon’s information index (Lewontin 1972); PIC, Polymorphism information content; Obs_Hom, Observed homozygosity.

### Admixture model–based population clusters and substructuring

**Figure 1:**
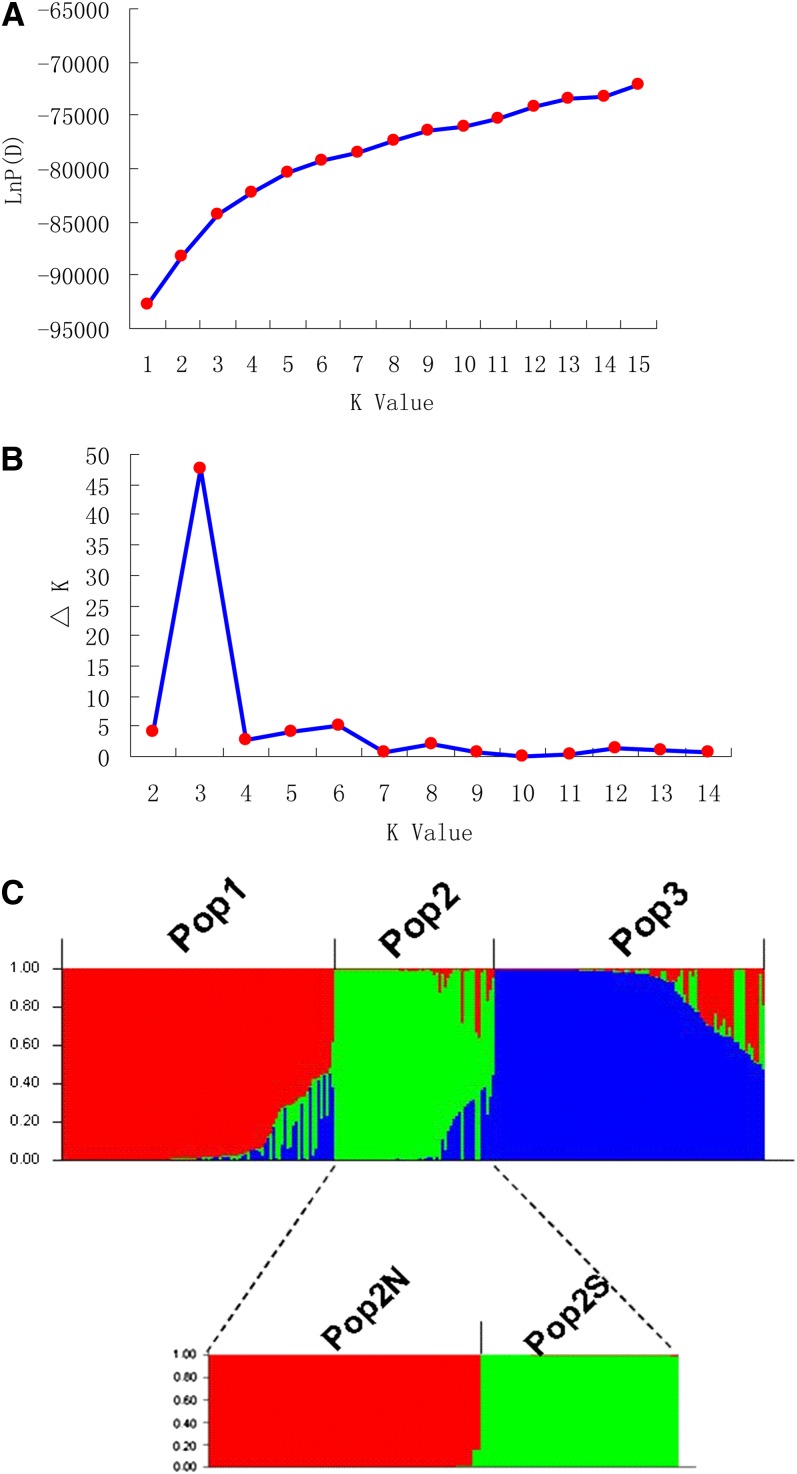
The two different methods for determining optimal value of K and inferred population structure of Chinese foxtail millet landrace accessions. (A) The ad hoc procedure described by Pritchard *et al.* (2000). (B) The second order of statistics (△K) based on Evanno *et al.* (2005). (C) Optimal population structure (K = 3) and substructuring (K = 2) of Pop2. Each landrace is represented by a single vertical line, each color represents one cluster or subpopulations, and the length of the colored segment shows the landrace’s estimated proportion of membership in that cluster as calculated by STRUCTURE.

Admixture model–based simulations were carried out by varying K from 1 to 15 with 20 iterations per K. When we ran the STRUCTURE simulation using all 250 accessions, the LnP(D) value increased with K from 1 to 15, but showed evident knees at k = 3 (Figure 1A). This implied that there might be three divergent subpopulations. According to the second-order statistics developed for STRUCTURE (Evanno *et al.* 2005) to assess number of subpopulations, the optimal value of K = 3 whose △K showed a peak was identified (Figure 1B). This suggested that these foxtail millet accessions can be grouped into three populations, as inferred from the model, here designated as Pop1 to Pop3, respectively (Figure 1C, top part). For each inferred population, substructuring under the topmost hierarchy was detected using a similar approach. Only Pop2 was clearly and conservatively divided into two (K = 2) subgroups among 20 iterations for each K (Figure S2). Finally, four parts were identified in all. This population structure is identical to the four topmost clusters when K was set as 4 (data not shown) using all 250 accessions. Therefore, the two subgroups under Pop2 were labeled as Pop2N and Pop2S, according to the geographical origins from the north and the south of sampled accessions. Pop1 includes 96 samples, collected mainly from the summer and spring sowing region (SSSR) located in the North China Plain and its closely joined regions, such as Liaoning, Jilin, Hebei, Beijing, Tianjin, Henan, Shandong, and Jiangsu provinces. Pop2N includes 33 lines mainly collected from the early spring sowing region (ESR) of Heilongjiang province in Northeast China Plain, which represents the most northerly part of foxtail millet cultivation. Pop2S includes 24 landraces, collected mainly from Southern China (SCR). Pop3 includes 97 samples, collected mostly from the spring sowing region (SR) of the inland Northwest and Inner Mongolia plateau. Non-admixture model–based simulations were also carried out (data not shown), and similar results were obtained.

**Figure 2:**
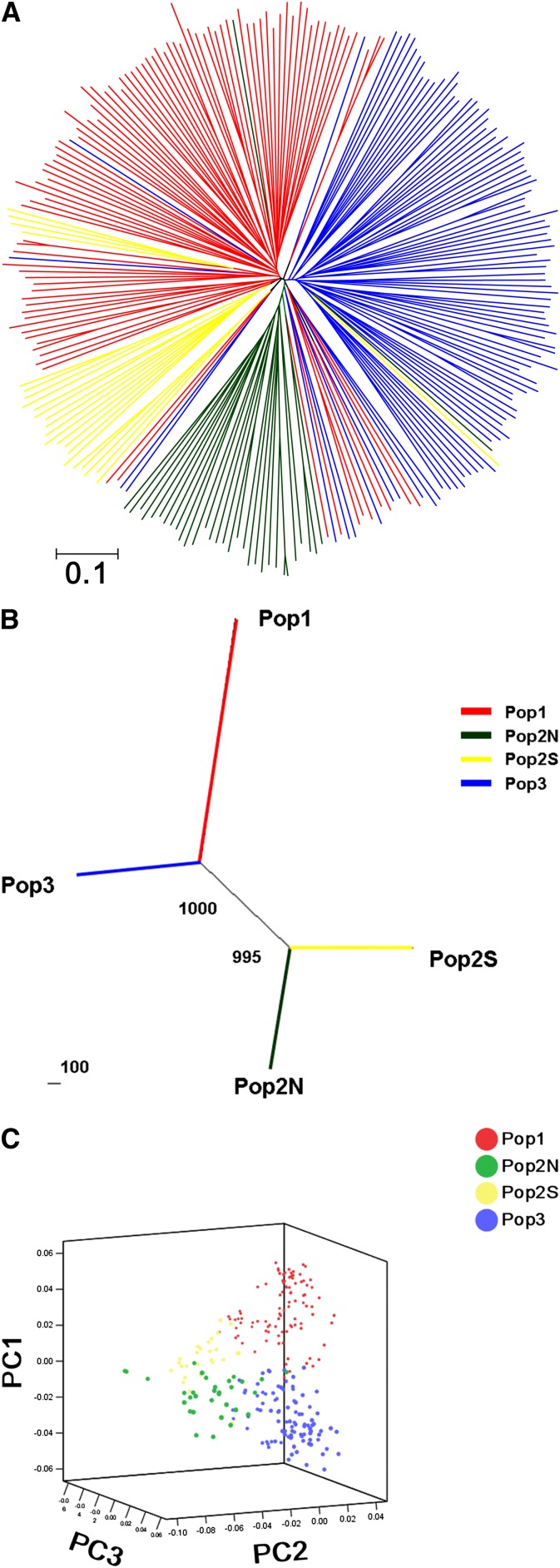
Neighbor-jointing (NJ) analysis and principal component analysis (PCA) of Chinese foxtail millet landraces. (A) Unrooted neighbor-jointing tree of 250 landraces; each colored branch represents one accession collected from corresponding inferred subpopulation. (B) NJ tree of inferred subgroups based on Nei’s genetic distance; bootstrap value (out of 1000) is indicated at the branch point. (C) Differentiation of genotypes from subpopulations based on the first three principal components derived from 77 SSR markers diversity analysis.

A neighbor-joining tree of 250 accessions was constructed by Nei’s (1983) genetic distance (Figure 2, A and B), which revealed genetic relationships that were fairly consistent with the STRUCTURE-based membership assignment for most of the accessions. A few cases of mixed distribution of subgroup accessions into different clusters can be seen in Figure 2A, which implies possible germplasm exchange between different eco-geographical regions, *e.g.* collection exchanging among Pop1, Pop3, and Pop2N. A principal component analysis was conducted to further assess the population subdivisions identified using STRUCTURE (Figure 2C). The first principal component explained 28.37%, the second principal component explained 21.41%, and the third principal component explained 15.58% of the molecular marker variation among 250 accessions. Plotting of the first three principal components shows clear separation of inferred subpopulations, which were highly consistent with the STRUCTURE and neighbor-joining analysis above.

Relationships among subpopulations inferred from STRUCTURE were identified by pairwise genetic distance and *Fst* analysis (Table 4). The genetic distance ranged from 0.2095 between Pop1 and Pop3 to 0.4003 between Pop2N and Pop2S, with an average of 0.3223. Pairwise *Fst* values for subpopulations ranged from 2.1345 between Pop1 and Pop2N to 2.7991 between Pop1 and Pop3, with an average of 2.3693. The genetic distance agrees with the trends of *Fst* estimates. For instance, higher genetic distance and *Fst* were found between Pop2N and Pop2S. The grouping relationships of all four subpopulations suggested by Table 4 were also largely concordant with the neighbor-joining analysis including all 250 accessions.

**Figure 3:**
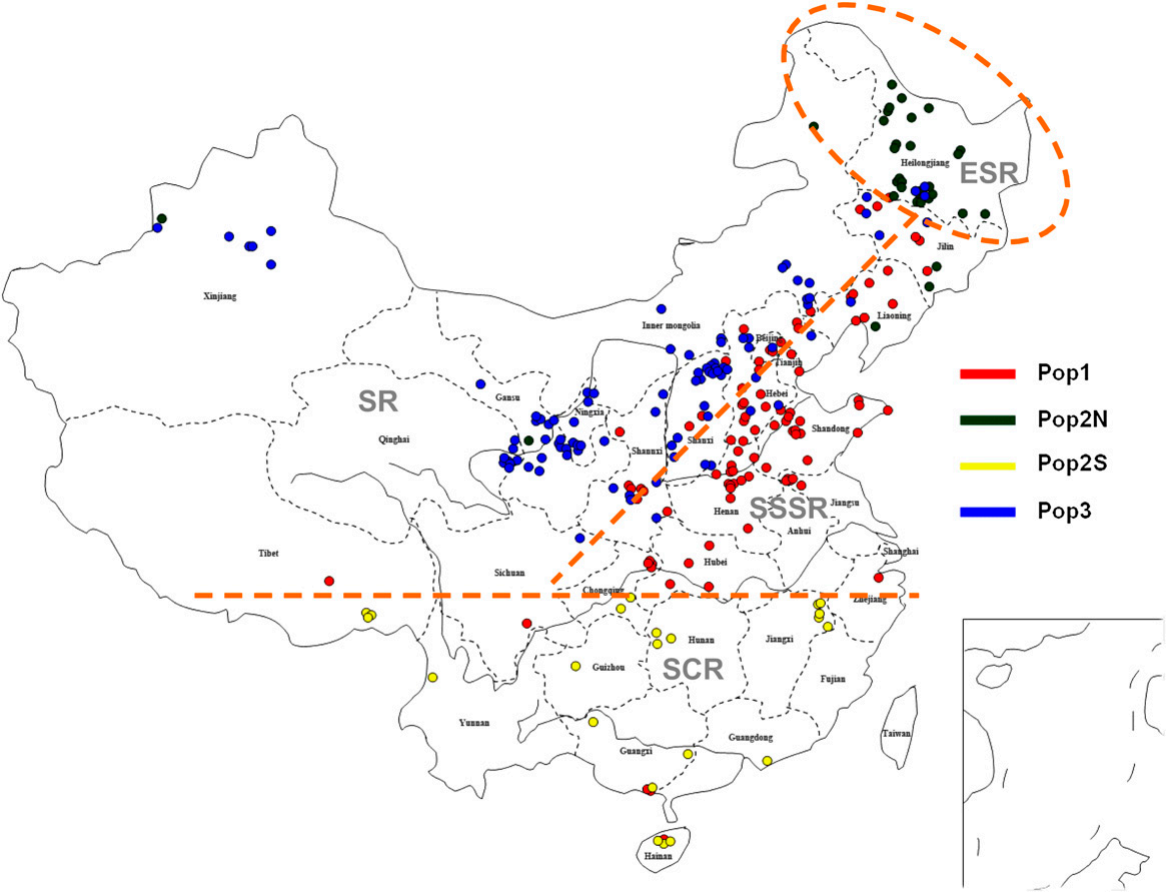
Map of the collection locations of Chinese foxtail millet landraces grouped by four subpopulations inferred in the investigations. Diverse color spots represent individuals from the various subpopulations. The four eco-regions in China for foxtail millet are illustrated by orange circles and lines.

To interpret the separation among subpopulations identified above, we depicted the habitat and geographical location from where each accession was collected (Figure 3), which revealed patterns of ecological differentiation that corresponded to the genetic subpopulations. Each subpopulation has a specific region. Pop1 was collected from the SSSR eco-region, Pop2N spreads in the ESR eco-region in Heilongjiang province, Pop2S contains individuals just from SCR eco-region in Southern China, Pop3 located mostly in the Loess Plateau and Inner Mongolia Plateau, widely distributed in SR eco-region in Northwest China.

### Genetic diversities of subpopulations

The genetic diversity was assessed per locus for each subpopulation (Table 3). Pop3 had the highest gene diversity, the highest number of alleles per locus, and the highest population-specific allele number, followed by Pop1. Among the 1612 alleles detected in the total populations, 514 (31.89%) were subpopulation-specific or private alleles. Pop2N and Pop2S had the lowest genetic diversity identified in this research. However, the sample size of Pop2N (33 accessions) is larger than Pop2S (24 accessions), indicating that Pop2N had the lower genetic diversity than Pop2S, which may be due to the short history and limited cultivated area of foxtail millet production in Heilongjiang. Pop2S, which had a relatively small number of samples, exhibited relatively high gene diversity, suggesting that there is a lot of genetic variation in landraces collected from Southern China.

In our analysis (Table 6), 6.17% of total genetic variance was explained among subpopulations, 0.99% was explained within individuals, and the remaining 92.83% was explained within subpopulations. Pop3 has the largest genetic variance (36.66%) among all subpopulations, followed by Pop1 (36.06%), Pop2N (11.37%), and Pop2S (8.74%).

**Table 6:** AMOVA for inferred subpopulations

Source of Variations		Sum of Variances	Percentage
Within populations	Pop1	11854.7325	0.3606
	Pop2N	3737.4325	0.1137
	Pop2S	2874.5608	0.0874
	Pop3	12050.7434	0.3666
Within individuals	Pop1	141.0000	0.0043
	Pop2N	54.0000	0.0016
	Pop2S	26.0000	0.0008
	Pop3	104.0000	0.0032
Among populations		2028.5863	0.0617
Total		32871.0556	1.0000

### LD among SSR loci

The extent of LD was assessed among 2844 marker pairs for all accessions as well as for subpopulations separately (Table 7). Across all accessions, as many as 56.33% of the total SSRs pairs were in LD (*P* < 0.05) after Bonferroni correction. For these loci pairs that had significant LD, D’ ranged from 0.174 to 0.948, with a mean of 0.426, and r^2^ ranged from 0.003 to 0.282, with a mean of 0.007. The frequency of pairs of loci with significant LD was reduced by more than half when LD was calculated within each subpopulation. The largest percentage (19.94%) of locus pairs in LD was found in Pop3 (accessions from SR eco-region), whereas Pop2N had the lowest percentage (13.95%) (landraces from ESR eco-region). Values of D’ and r^2^ was increased when analyzed within each subpopulations. Pop2S presented the highest mean value for D’ of 0.808 and r^2^ of 0.077, and the lowest mean value of D’ and r^2^ were found in Pop1 as 0.536 and 0.017, respectively.

**Table 7:** Percentage of SSR locus pairs in significant (*P* < 0.05) LD and LD statistics D’ and r^2^ of Chinese foxtail millet landrace populations

	No. of Significant Marker Pairs in LD	No. of Marker Pairs Evaluated	Fraction of Locus Pairs (%)	Extent of LD	
				D’	r^2^
Pop1	537	2836	18.94	0.536	0.017
Pop2N	382	2739	13.95	0.719	0.057
Pop2S	484	2774	17.45	0.808	0.077
Pop3	575	2884	19.94	0.560	0.017
All	1602	2844	56.33	0.426	0.007

**Figure 4:**
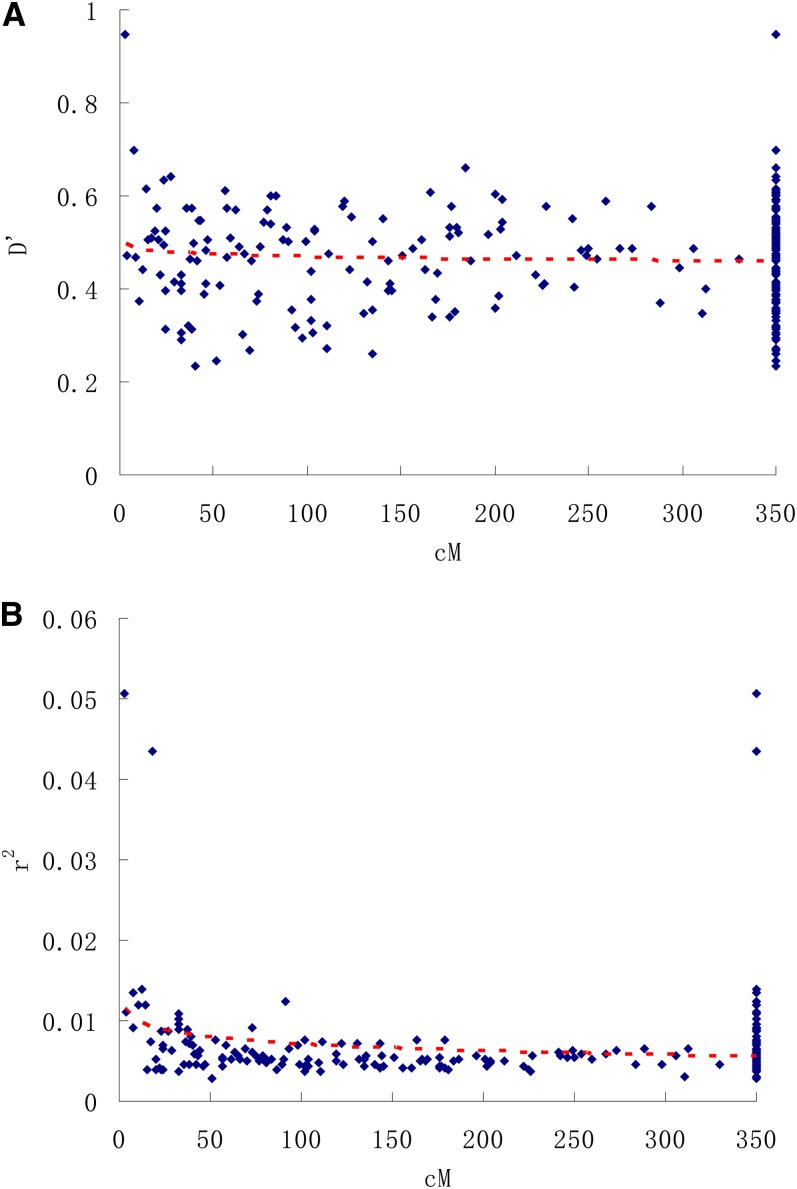
Scatterplot of linkage disequilibrium (LD) statistics D’ (A) and r^2^ (B) between SSR pairs *vs.* intermarker genetic distance (cM) for the whole population. The observed values for interchromosomal markers are compiled in a single file at 350 cM.

The distribution of data points in the plot of LD (Figure 4) decay against genetic linkage distance (cM) within the nine chromosomes showed that LD was not a simple monotonic function of the distance between markers, especially for r^2^. LD was common for distances less than 20 cM, and occasionally, LD occurred between SSR loci that were farther apart. The average distance of LD decay identified in foxtail millet landraces was 15∼20 cM from our data.

## Discussion

### High molecular diversity of Chinese foxtail millet landraces

In this investigation, a set of foxtail millet landrace lines, which represent 1% of the Chinese National Gene Bank accessions, was characterized using SSRs. The average number of alleles per locus was 20.9, which was higher than Jia’s result of 6.16 alleles per loci (Jia *et al.* 2009), illustrated that Chinese foxtail millet landraces contain high genetic diversity, which could provide important gene resources for foxtail millet breeding programs and for functional genomics study. Foxtail millet is being developed as a model organism for functional genomics studies on plant morphology, physiology (Doust *et al.* 2009) and C_4_ photosynthesis (Brutnell *et al.* 2010) owing to its small genome and inbreeding nature. Higher genetic diversity of landraces is favorable for genetic marker development, construction of segregating populations, functional gene cloning and association mapping. High diversity also provides enriched gene resources for gene mining in the grass family.

In ancient north and northeast China, foxtail millet has been planted as a staple food for thousands of years, and varieties of different ecotypes were developed by natural and artificial selection (Li and Wu 1996). Given the diversity of eco-geographical conditions and the long history of foxtail millet cultivation in China, it is not surprising that such high levels of diversity have evolved. Related investigations on genetic diversity of foxtail millet landraces based on AFLP markers (Ennequin *et al.* 2000) also suggest that Chinese accessions have the highest diversity compared with accessions from other countries. Moreover, high Chinese foxtail millet diversity was in good accordance with single gene studies, such as on *Waxy* (Van *et al.* 2008). Thus, further trials to establish relative genetic diversity should be carried out on a worldwide set of accessions and on developed elite cultivars in the future.

### Genetic structure of Chinese foxtail millet landrace concordance with geographical ecotypes

Structure-based subpopulations inferred in this research were consistent with ecotypes and eco-regions of foxtail millet landrace distribution in China. A detailed set of 11 eco-regions of foxtail millet have been classified (Wang and Guo 1997) that can be summarized into the four main eco-regions of foxtail millet landrace distribution: (1) early spring sowing region (ESR) in the northeast Heilongjiang province, where foxtail millet was sowed in late April or early May annually (if sown later than early May, plants cannot reach maturity due to the cold weather); (2) spring sowing region (SR), including Shanxi, Shannxi, Inner Mongolia, Gansu, Ningxia, Xianjiang, and western part of Jilin and Liaoning provinces, where foxtail millet was sowed in May; (3) summer and spring sowing region (SSSR), including Henan, Hebei, Shandong, Beijing, Tianjin, and connected parts of Jiangsu, Liaoning and Jilin provinces, where foxtail millet can be sowed from late April to early July; and (4) Southern China region (SCR), including all provinces south of Qinling Mountain, where foxtail millet can be sowed even in late summer and early autumn.

According to the topmost hierarchy structure inferred by this study, three clusters were identified and two (Pop1 and Pop3) of them were consistent with eco-origins classified previously by Wang and Guo (1997). Pop2 includes accessions collected both from ESR and SCR eco-regions; this may due to several unclear reasons, such as unequal distribution density of SSR markers used in this study through nine chromosomes of foxtail millet and effect of LD among SSR loci, on the result of model-based simulation. After substructuring of the three topmost clusters, only Pop2 was conservatively divided into two parts (Pop2N and Pop2S) through different iterations, which is identical with the two eco-regions (ESR and SCR) where samples were collected. The limited number of SSR markers is the main challenge of evaluating population structure of foxtail millet accessions. In this study, all 77 SSR loci were used for STRUCTURE analysis, neglecting potential linkage and LD effects between the loci, which should be considered and evaluated in following studies based on more SSR markers.

Nearly all landraces of Pop2N come from ESR; these landraces are adapted to the cold weather and short growth period in the most northern part of China and are usually very sensitive to both temperature and light change (Wang and Guo 1997). Accessions of Pop3 were collected mostly from the Loess plateau and Inner Mongolia plateau, and they are adapted to long periods of sunshine in low latitudes and lower temperatures in high altitudes, such that landraces of these groups are mostly insensitive to light changes but sensitive or medium-sensitive to temperature changes (Wang and Guo 1997). Varieties in SR regions are usually robust with long panicles. Adapted to high temperatures and plentiful sunlight, accessions in Pop1 from the SSSR regions usually are small in stature with short panicles, and they are less sensitive to both temperature and sunlight change. Accessions in Pop2S mostly come from Southern China and are adapted to humidity and high temperatures. The low level of mixture detected from the NJ diagram (Figure S1), indicating that a few landraces from certain eco-regions may be classified into subgroups from other eco-regions, suggests frequent variety exchange.

### Foxtail millet was probably domesticated in the Yellow River regions

Foxtail millet is one of the oldest cereals in Eurasia. Its origin and domestication have always been interesting because of its great contribution to ancient civilization. China has been identified as one of the main original and diversity centers for foxtail millet (Vavilov 1926), later supported by related studies on morphological diversity (Li *et al.* 1995; Ochiai 1996), isozymes type (Croullebois *et al.* 1989), and recent archeological discoveries (Lu *et al.* 2009; Barton *et al.* 2009). But where foxtail millet was domesticated and how many times it was domesticated in China still need to be answered. The region with the highest genetic diversity is generally considered as the center of origin of a species (Vavilov 1951; Chang 1989; Dong *et al.* 2004). Among the subpopulations, Pop3 has the highest allelic richness and the largest number of cluster-specific alleles, followed by Pop1 and Pop2. Pop3 also presents the highest gene diversity among all subpopulations, followed by Pop1 as the second, which may suggest that foxtail millet was probably domesticated in regions where Pop3 and Pop1 are located. Pop1 is located in the downstream regions of Yellow River, and Pop3 is located in the middle-stream regions of the Yellow River, so foxtail millet was likely domesticated in the Yellow River regions and then spread to other parts of the county. This hypothesis is supported by the earliest archeological evidence of foxtail millet domestication found in the Yellow River regions (Barton *et al.* 2009; Lu *et al.* 2009), which date back more than 8700 years.

AMOVA analysis implies that two centers of diversity exist in China, one of which is the spring sowing region and the other is the summer- and spring-sowing region (Table 6). This conjecture was supported not only by the large number of population-specific accessions but also by high gene diversity and PIC values of these two eco-regions. Recognizing two foxtail millet diversity centers in China is important to germplasm management, breeding and basic genomic studies. However, whether these two diversity centers means two independent domestication events in China still needs further research.

### Linkage disequilibrium in Chinese foxtail millet landrace

SSR markers have been used for primary evaluation of LD across the genome of crops, including rice (Jin *et al.* 2010), maize (Wang *et al.* 2008), and soybean (Li *et al.* 2008). As a self-pollinating species, foxtail millet might be expected to have a high level of LD, but Van *et al.* (2008) detected low levels of LD by analysis of molecular differences of the *Waxy* gene in 113 worldwide foxtail millet landraces, and Wang *et al.* (2010) detected low levels of LD when examining nine fragmental genomic sequences from 50 foxtail millet landraces. In our research using SSRs with Chinese foxtail landraces, levels of LD were also lower than those identified by studies on other self-pollinating crops. This result reflects a relatively low level of LD and presumably limits sizes of haplotypes in foxtail millet landraces.

Population size and pollinating behavior may influence extent of LD. In cross-pollinating crops like maize, LD diminished even after only 2 Kb of genomic sequence (Remington *et al.* 2001). Similar patterns were observed in sugar beet (*Beta vulgaris*), whose LD extended only to 3 cM (Kraft *et al.* 2000). However, in self-pollinating crops such as barley, LD commonly extends for distances of up to 10 cM (Kraakman *et al.* 2004), and in some *Arabidopsis* populations, LD exceeds 50 cM (Nordborg *et al.* 2002). LD is high in rice, and LD decay values are 20∼30 cM (Agrama *et al.* 2007). In terms of this investigation, we observed LD decay in less than 20 cM of genetic distance in foxtail millet landrace genome, suggesting that it could be possible to achieve resolution down to the 20 cM level for association mapping in foxtail millet, which is similar to the situation in rice.

## Supplementary Material

Supporting Information
