# Supplementary material for: Genetic Diversity and Population Structure of Chinese Foxtail Millet [Setaria italica (L.) Beauv.] Landraces
Source: G3 (Bethesda). 2012 Jul 1;2(7):769–77. doi: 10.1534/g3.112.002907 (PMC3385983; doi:10.1534/g3.112.002907)
Supplement: Supporting Information [file supp_2_7_769__index.html]

Supporting Information 

# Genetic Diversity and Population Structure of Chinese Foxtail Millet [*Setaria italica* (L.) Beauv.] Landraces

## Supporting Information for Wang *et al.*, 2012

**Files in this Data Supplement:**

- Supporting Information - (PDF, 237 KB)
- Figure S1 - NJ tree of 250 Chinese foxtail millet landraces (PDF, 159 KB)
- Figure S2 - Determination of optimal value of K for substructuring of inferred Pop2 (PDF, 79 KB)
- Table S1 - Genetic diversity identified for 77 SSR markers in 250 landraces (PDF, 82 KB)
- Table S2 - New SSR markers used in this investigation (PDF, 61 KB)
