# Supplementary material for: Genetic Diversity and Population Structure of Chinese Foxtail Millet [Setaria italica (L.) Beauv.] Landraces
Source: G3 (Bethesda). 2012 Jul 1;2(7):769–77. doi: 10.1534/g3.112.002907 (PMC3385983; doi:10.1534/g3.112.002907)
Supplement: Supporting Information [file supp_2.7.769_002907SI.pdf]

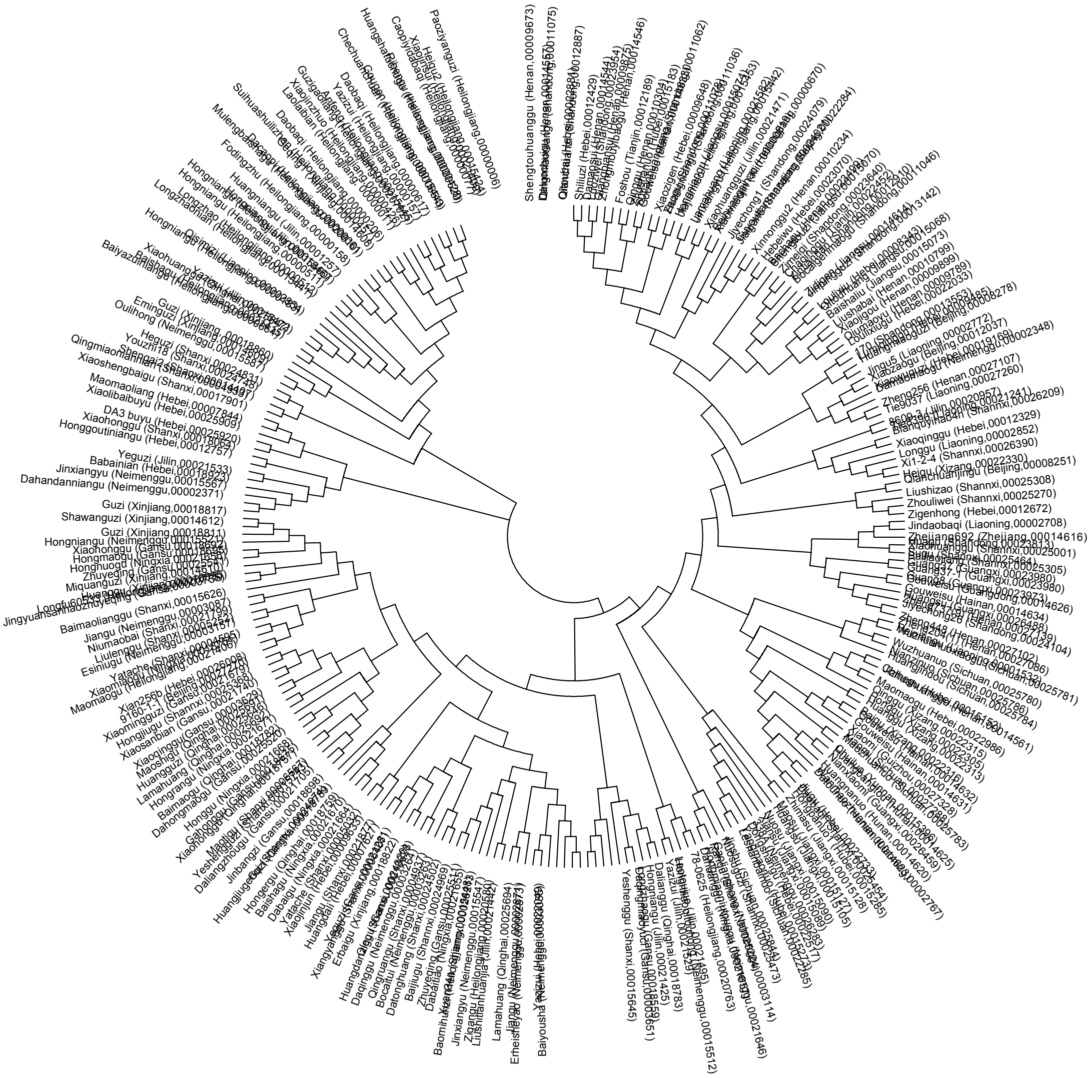

Figure S1 NJ tree of 250 Chinese foxtail millet landraces. Each accession was displayed with name, origin provinces and register number in Chinese National Gene Bank (CNGB).

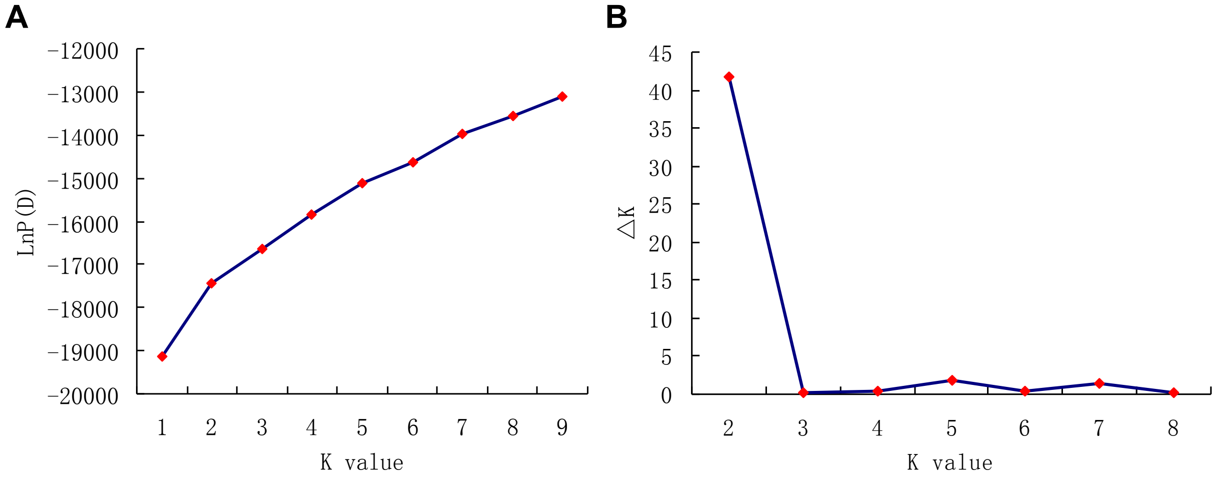

Figure S2 Determination of optimal value of K for substructuring of inferred Pop2. Length of burn-in of the Markov Chain Monte Carlo (MCMC) iterations was set to 100,000 and data were collected over 100,000 MCMC iterations in each run. 20 iterations per K (1 to 9) were conducted. **(A)** The ad hoc procedure described by PRITCHARD *et al*. (2000) ; **(B)**The second order of statistics (ΔK) based on EVANNO *et al.* (2005).

Table S1 Genetic diversity identified for 77 SSR markers in 250 landraces

Marker	Major.Allele. Frquency	Genotype No	Allele No	Ne	Shannon's I	Gene Diversity	Heterozy -gosity	PIC
**B1**	0.3140	16.0000	15.0000	5.6337	2.0112	0.8220	0.0040	0.8018
**b163**	0.2880	19.0000	16.0000	6.7597	2.2115	0.8511	0.0120	0.8369
**b165**	0.1120	31.0000	28.0000	15.0006	2.9132	0.9338	0.0160	0.9299
**b186**	0.1320	31.0000	31.0000	17.6056	3.0955	0.9432	0.0000	0.9404
**b217**	0.0880	29.0000	28.0000	18.1081	3.0287	0.9448	0.0040	0.9420
**b218**	0.1290	26.0000	22.0000	12.1393	2.6809	0.9176	0.0161	0.9117
**b225**	0.1386	21.0000	17.0000	12.0542	2.6207	0.9170	0.0161	0.9111
**b237**	0.1800	16.0000	13.0000	8.0959	2.2005	0.8765	0.0120	0.8637
**b258**	0.3373	34.0000	31.0000	6.8245	2.6024	0.8533	0.0161	0.8450
**b263**	0.3200	26.0000	25.0000	6.2164	2.3247	0.8397	0.0040	0.8258
**b269**	0.1100	34.0000	32.0000	15.053	2.9802	0.9336	0.0080	0.9297
**p3**	0.1400	37.0000	35.0000	18.307	3.1678	0.9454	0.0080	0.9429
**p10**	0.2760	24.0000	21.0000	6.0878	2.1625	0.8358	0.0120	0.8170
**p29**	0.1640	23.0000	22.0000	10.3778	2.5771	0.9036	0.0040	0.8959
**p32**	0.5440	16.0000	14.0000	2.9208	1.5379	0.6573	0.0080	0.6286
**p42**	0.2580	14.0000	11.0000	5.9626	1.9898	0.8318	0.0120	0.8117
**p44**	0.2460	21.0000	20.0000	6.4476	2.1881	0.8458	0.0040	0.8289
**x4**	0.4480	14.0000	11.0000	2.9717	1.3459	0.6620	0.0200	0.6038
**x298**	0.3520	15.0000	13.0000	4.2397	1.7073	0.7641	0.0080	0.7298
**p33**	0.3560	10.0000	9.0000	3.7339	1.4952	0.7414	0.0080	0.7002
**p2**	0.1310	30.0000	27.0000	13.6873	2.8744	0.9269	0.0161	0.9224
**b102**	0.1180	48.0000	45.0000	17.313	3.2287	0.9421	0.0160	0.9392
**b174**	0.1480	29.0000	27.0000	14.3086	2.8866	0.9302	0.0120	0.9261
**p20**	0.2680	16.0000	16.0000	7.2071	2.2458	0.8612	0.0000	0.8481
**b171**	0.1260	33.0000	32.0000	16.4582	3.0463	0.9392	0.0040	0.9360
**b109**	0.1940	40.0000	37.0000	13.926	3.1096	0.9303	0.0120	0.9270

Marker	Major.Allele. Frquency	Genotype No	Allele No	Ne	Shannon's I	Gene Diversity	Heterozy -gosity	PIC
**b159**	0.1240	18.0000	18.0000	11.3389	2.5499	0.9118	0.0000	0.9050
**b266**	0.3240	12.0000	11.0000	4.2337	1.688	0.7638	0.0040	0.7279
**p45**	0.3220	12.0000	10.0000	3.5792	1.432	0.7206	0.0080	0.6683
**p41**	0.2540	24.0000	21.0000	8.3012	2.4388	0.8795	0.0120	0.8697
**p58**	0.5580	21.0000	19.0000	2.977	1.7234	0.6641	0.0080	0.6483
**p59**	0.2280	17.0000	17.0000	8.4551	2.3621	0.8817	0.0000	0.8712
**p61**	0.2680	18.0000	16.0000	7.0193	2.2511	0.8575	0.0080	0.8436
**p80**	0.1280	33.0000	31.0000	13.5914	2.8771	0.9264	0.0080	0.9217
**p88**	0.1960	23.0000	21.0000	8.9063	2.4859	0.8921	0.0080	0.8834
**p98**	0.2720	9.0000	9.0000	4.488	1.6152	0.7772	0.0000	0.7406
**p100**	0.4320	7.0000	7.0000	3.4295	1.4077	0.7084	0.0000	0.6632
**p17x**	0.4060	13.0000	11.0000	3.4823	1.4991	0.7128	0.0080	0.6669
**p4**	0.1820	35.0000	32.0000	10.23	2.7674	0.9022	0.0120	0.8950
**b247**	0.2220	29.0000	26.0000	9.66	2.5957	0.8965	0.0280	0.8887
**b241**	0.0800	31.0000	26.0000	17.5439	2.9988	0.9430	0.0200	0.9400
**b153**	0.1900	24.0000	18.0000	8.912	2.3868	0.8878	0.0280	0.8776
**b185**	0.1025	34.0000	26.0000	16.6116	2.9526	0.9398	0.0328	0.9365
**b101**	0.1154	36.0000	31.0000	17.3543	3.0691	0.9424	0.0243	0.9394
**b123**	0.2420	22.0000	18.0000	6.4228	2.1956	0.8443	0.0160	0.8274
**b126**	0.2520	16.0000	14.0000	6.0759	2.0744	0.8354	0.0080	0.8162
**b145**	0.4840	9.0000	8.0000	3.0455	1.3196	0.6716	0.0080	0.6229
**b166**	0.1180	27.0000	21.0000	14.1868	2.7997	0.9295	0.0240	0.9251
**b169**	0.1240	25.0000	23.0000	13.6806	2.8138	0.9269	0.0080	0.9222
**b200**	0.1600	21.0000	19.0000	10.8989	2.5899	0.9082	0.0080	0.9012
**b224**	0.0900	36.0000	32.0000	22.0497	3.2309	0.9547	0.0160	0.9528
**b236**	0.1120	40.0000	37.0000	20.5965	3.2353	0.9514	0.0120	0.9493
**b242**	0.1280	26.0000	25.0000	12.7694	2.7279	0.9217	0.0200	0.9163

Marker	Major.Allele. Frquency	Genotype No	Allele No	Ne	Shannon's I	Gene Diversity	Heterozy -gosity	PIC
**b246**	0.0860	41.0000	27.0000	17.7607	3.0099	0.9439	0.0560	0.9410
**b187**	0.1080	24.0000	23.0000	14.9575	2.8457	0.9331	0.0040	0.9291
**p14**	0.2041	33.0000	23.0000	8.9516	2.5322	0.8883	0.0449	0.8790
**b223**	0.1245	30.0000	25.0000	14.7026	2.8574	0.9320	0.0201	0.9279
**p16**	0.2880	13.0000	11.0000	5.6697	1.9057	0.8236	0.0080	0.8015
**p8**	0.2177	19.0000	18.0000	9.5407	2.5288	0.8952	0.0040	0.8871
**b117**	0.3340	29.0000	20.0000	5.4322	2.0891	0.8148	0.0600	0.7953
**b180**	0.2843	16.0000	11.0000	5.2891	1.8223	0.8123	0.0282	0.7871
**b181**	0.3233	13.0000	11.0000	4.995	1.8329	0.7998	0.0120	0.7737
**b182**	0.1980	42.0000	35.0000	9.1148	2.6825	0.8906	0.0360	0.8819
**b189**	0.0858	35.0000	29.0000	18.1856	3.0633	0.9475	0.0293	0.9450
**MPGA50**	0.2530	21.0000	17.0000	6.9911	2.2206	0.8573	0.0201	0.8428
**b249**	0.1000	45.0000	33.0000	17.1987	3.0247	0.9426	0.0680	0.9396
**MPGA31**	0.4538	25.0000	16.0000	3.9383	1.8537	0.7497	0.0402	0.7301
**MPGD13**	0.5100	6.0000	6.0000	2.7102	1.1328	0.6310	0.0000	0.5684
**MPGD32**	0.5442	14.0000	8.0000	2.8204	1.3821	0.6509	0.0402	0.6179
**p89**	0.1280	40.0000	28.0000	14.3135	2.8914	0.9305	0.0600	0.9263
**p78**	0.1774	27.0000	19.0000	9.6484	2.4748	0.8952	0.0444	0.8863
**MPGD44**	0.5665	12.0000	8.0000	2.6732	1.3366	0.6279	0.0282	0.5937
**si017**	0.2533	31.0000	25.0000	9.1202	2.5863	0.8904	0.0262	0.8827
**si119**	0.3557	15.0000	11.0000	5.0485	1.8976	0.8019	0.0163	0.7801
**si132**	0.2860	19.0000	14.0000	5.4134	1.917	0.8152	0.0240	0.7914
**si227**	0.1928	23.0000	16.0000	7.5212	2.2133	0.8670	0.0321	0.8529
**b250**	0.0685	46.0000	41.0000	23.8019	3.3637	0.9580	0.0242	0.9563

Table S2 New SSR markers used in this investigation

Serial no.	Name of SSR marker	Primer sequence	Repeat type	Chromosoe located	Tm (℃)	Expected size (bp)
1	Si017	F：CCCAGTCACGACGTTGCCGTCGTGCTAGGATACGTT<br>R：AAGGTTGGTAGTTGCCCTTG	(CA)27	5	60	298
2	Si227	F：CCCAGTCACGACGTTGGCGAGCTATACCTCTCACCG<br>R：TGATGGAGCAGCAAGAAATG	(GA)10	8	60	183
3	Si119	F：GCTGAGAAAGTTTGTTGGGC<br>R：GAATTCGAACCGAGCACATT	(AG)18	9	60	293
4	Si132	F：CCCAGTCACGACGTTGCAAATTCTCACCAATGCCCT<br>R：GTTCAGTGGTTGCAAAGGGT	(CA)16	9	60	375
5	B1 (AGDZ23-35)	F: GTTGTCCCTTTCCTCTGTGCTTT<br>R: ATCTCCCGGCGCTTGCTT	(AG)35		53	258
6	X4	F:GTGATGCCACGACACCATAG<br>R: CCTGTTCTTCATCACTGCGA	Dinucleotide		58	313
7	X298	F: CAGAGAGGCATAACGCATGA<br>R: TAGACAACCGATGCAAGCAG	Dinucleotide	9	58	339
