# Supplementary material for: Genetic Diversity and Population Structure of Chinese Foxtail Millet [Setaria italica (L.) Beauv.] Landraces
Source: G3 (Bethesda). 2012 Jul 1;2(7):769–77. doi: 10.1534/g3.112.002907 (PMC3385983; doi:10.1534/g3.112.002907)
Supplement: Supporting Information [file supp_2.7.769_FigureS1.pdf]

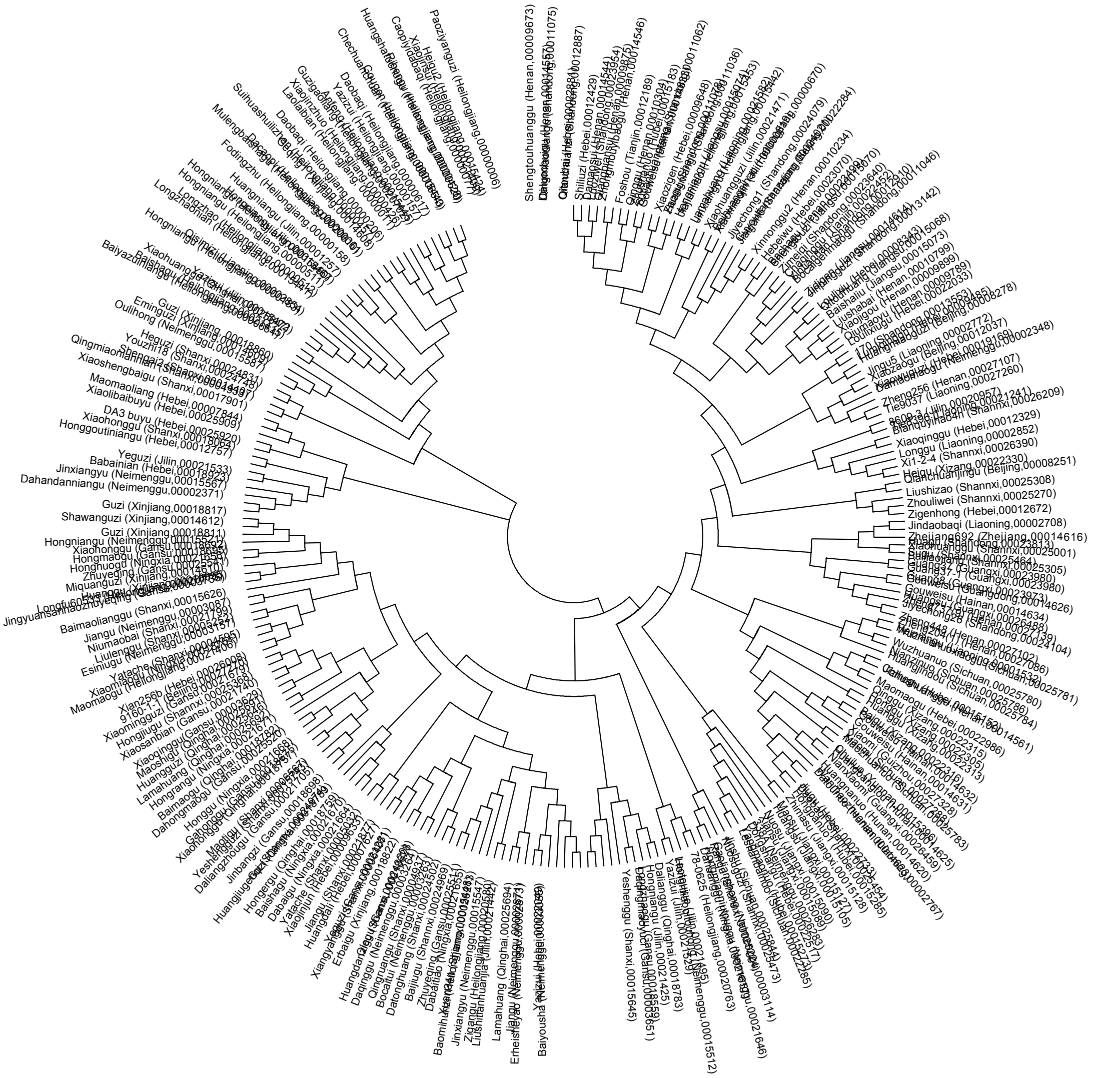

Figure S1 NJ tree of 250 Chinese foxtail millet landraces. Each accession was displayed with name, origin provinces and register number in Chinese National Gene Bank (CNGB).
