# Supplementary material for: Genetic Diversity and Population Structure of Chinese Foxtail Millet [Setaria italica (L.) Beauv.] Landraces
Source: G3 (Bethesda). 2012 Jul 1;2(7):769–77. doi: 10.1534/g3.112.002907 (PMC3385983; doi:10.1534/g3.112.002907)
Supplement: Supporting Information [file supp_2.7.769_FigureS2.pdf]

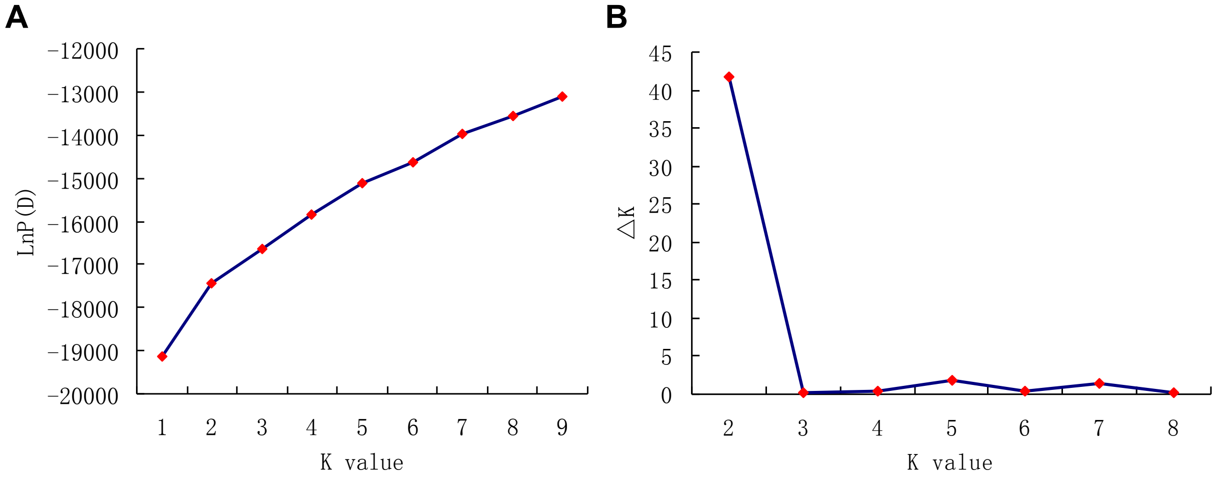

Figure S2 Determination of optimal value of K for substructuring of inferred Pop2. Length of burn-in of the Markov Chain Monte Carlo (MCMC) iterations was set to 100,000 and data were collected over 100,000 MCMC iterations in each run. 20 iterations per K (1 to 9) were conducted. **(A)** The ad hoc procedure described by PRITCHARD *et al*. (2000) ; **(B)**The second order of statistics (ΔK) based on EVANNO *et al.* (2005).
