# Supplementary material for: Genetic Diversity and Population Structure of Chinese Foxtail Millet [Setaria italica (L.) Beauv.] Landraces
Source: G3 (Bethesda). 2012 Jul 1;2(7):769–77. doi: 10.1534/g3.112.002907 (PMC3385983; doi:10.1534/g3.112.002907)
Supplement: Supporting Information [file supp_2.7.769_TableS1.pdf]

Table S1 Genetic diversity identified for 77 SSR markers in 250 landraces

Marker	Major.Allele. Frquency	Genotype No	Allele No	Ne	Shannon's I	Gene Diversity	Heterozy -gosity	PIC
**B1**	0.3140	16.0000	15.0000	5.6337	2.0112	0.8220	0.0040	0.8018
**b163**	0.2880	19.0000	16.0000	6.7597	2.2115	0.8511	0.0120	0.8369
**b165**	0.1120	31.0000	28.0000	15.0006	2.9132	0.9338	0.0160	0.9299
**b186**	0.1320	31.0000	31.0000	17.6056	3.0955	0.9432	0.0000	0.9404
**b217**	0.0880	29.0000	28.0000	18.1081	3.0287	0.9448	0.0040	0.9420
**b218**	0.1290	26.0000	22.0000	12.1393	2.6809	0.9176	0.0161	0.9117
**b225**	0.1386	21.0000	17.0000	12.0542	2.6207	0.9170	0.0161	0.9111
**b237**	0.1800	16.0000	13.0000	8.0959	2.2005	0.8765	0.0120	0.8637
**b258**	0.3373	34.0000	31.0000	6.8245	2.6024	0.8533	0.0161	0.8450
**b263**	0.3200	26.0000	25.0000	6.2164	2.3247	0.8397	0.0040	0.8258
**b269**	0.1100	34.0000	32.0000	15.053	2.9802	0.9336	0.0080	0.9297
**p3**	0.1400	37.0000	35.0000	18.307	3.1678	0.9454	0.0080	0.9429
**p10**	0.2760	24.0000	21.0000	6.0878	2.1625	0.8358	0.0120	0.8170
**p29**	0.1640	23.0000	22.0000	10.3778	2.5771	0.9036	0.0040	0.8959
**p32**	0.5440	16.0000	14.0000	2.9208	1.5379	0.6573	0.0080	0.6286
**p42**	0.2580	14.0000	11.0000	5.9626	1.9898	0.8318	0.0120	0.8117
**p44**	0.2460	21.0000	20.0000	6.4476	2.1881	0.8458	0.0040	0.8289
**x4**	0.4480	14.0000	11.0000	2.9717	1.3459	0.6620	0.0200	0.6038
**x298**	0.3520	15.0000	13.0000	4.2397	1.7073	0.7641	0.0080	0.7298
**p33**	0.3560	10.0000	9.0000	3.7339	1.4952	0.7414	0.0080	0.7002
**p2**	0.1310	30.0000	27.0000	13.6873	2.8744	0.9269	0.0161	0.9224
**b102**	0.1180	48.0000	45.0000	17.313	3.2287	0.9421	0.0160	0.9392
**b174**	0.1480	29.0000	27.0000	14.3086	2.8866	0.9302	0.0120	0.9261
**p20**	0.2680	16.0000	16.0000	7.2071	2.2458	0.8612	0.0000	0.8481
**b171**	0.1260	33.0000	32.0000	16.4582	3.0463	0.9392	0.0040	0.9360
**b109**	0.1940	40.0000	37.0000	13.926	3.1096	0.9303	0.0120	0.9270

Marker	Major.Allele. Frquency	Genotype No	Allele No	Ne	Shannon's I	Gene Diversity	Heterozy -gosity	PIC
**b159**	0.1240	18.0000	18.0000	11.3389	2.5499	0.9118	0.0000	0.9050
**b266**	0.3240	12.0000	11.0000	4.2337	1.688	0.7638	0.0040	0.7279
**p45**	0.3220	12.0000	10.0000	3.5792	1.432	0.7206	0.0080	0.6683
**p41**	0.2540	24.0000	21.0000	8.3012	2.4388	0.8795	0.0120	0.8697
**p58**	0.5580	21.0000	19.0000	2.977	1.7234	0.6641	0.0080	0.6483
**p59**	0.2280	17.0000	17.0000	8.4551	2.3621	0.8817	0.0000	0.8712
**p61**	0.2680	18.0000	16.0000	7.0193	2.2511	0.8575	0.0080	0.8436
**p80**	0.1280	33.0000	31.0000	13.5914	2.8771	0.9264	0.0080	0.9217
**p88**	0.1960	23.0000	21.0000	8.9063	2.4859	0.8921	0.0080	0.8834
**p98**	0.2720	9.0000	9.0000	4.488	1.6152	0.7772	0.0000	0.7406
**p100**	0.4320	7.0000	7.0000	3.4295	1.4077	0.7084	0.0000	0.6632
**p17x**	0.4060	13.0000	11.0000	3.4823	1.4991	0.7128	0.0080	0.6669
**p4**	0.1820	35.0000	32.0000	10.23	2.7674	0.9022	0.0120	0.8950
**b247**	0.2220	29.0000	26.0000	9.66	2.5957	0.8965	0.0280	0.8887
**b241**	0.0800	31.0000	26.0000	17.5439	2.9988	0.9430	0.0200	0.9400
**b153**	0.1900	24.0000	18.0000	8.912	2.3868	0.8878	0.0280	0.8776
**b185**	0.1025	34.0000	26.0000	16.6116	2.9526	0.9398	0.0328	0.9365
**b101**	0.1154	36.0000	31.0000	17.3543	3.0691	0.9424	0.0243	0.9394
**b123**	0.2420	22.0000	18.0000	6.4228	2.1956	0.8443	0.0160	0.8274
**b126**	0.2520	16.0000	14.0000	6.0759	2.0744	0.8354	0.0080	0.8162
**b145**	0.4840	9.0000	8.0000	3.0455	1.3196	0.6716	0.0080	0.6229
**b166**	0.1180	27.0000	21.0000	14.1868	2.7997	0.9295	0.0240	0.9251
**b169**	0.1240	25.0000	23.0000	13.6806	2.8138	0.9269	0.0080	0.9222
**b200**	0.1600	21.0000	19.0000	10.8989	2.5899	0.9082	0.0080	0.9012
**b224**	0.0900	36.0000	32.0000	22.0497	3.2309	0.9547	0.0160	0.9528
**b236**	0.1120	40.0000	37.0000	20.5965	3.2353	0.9514	0.0120	0.9493
**b242**	0.1280	26.0000	25.0000	12.7694	2.7279	0.9217	0.0200	0.9163

Marker	Major.Allele. Frquency	Genotype No	Allele No	Ne	Shannon's I	Gene Diversity	Heterozy -gosity	PIC
**b246**	0.0860	41.0000	27.0000	17.7607	3.0099	0.9439	0.0560	0.9410
**b187**	0.1080	24.0000	23.0000	14.9575	2.8457	0.9331	0.0040	0.9291
**p14**	0.2041	33.0000	23.0000	8.9516	2.5322	0.8883	0.0449	0.8790
**b223**	0.1245	30.0000	25.0000	14.7026	2.8574	0.9320	0.0201	0.9279
**p16**	0.2880	13.0000	11.0000	5.6697	1.9057	0.8236	0.0080	0.8015
**p8**	0.2177	19.0000	18.0000	9.5407	2.5288	0.8952	0.0040	0.8871
**b117**	0.3340	29.0000	20.0000	5.4322	2.0891	0.8148	0.0600	0.7953
**b180**	0.2843	16.0000	11.0000	5.2891	1.8223	0.8123	0.0282	0.7871
**b181**	0.3233	13.0000	11.0000	4.995	1.8329	0.7998	0.0120	0.7737
**b182**	0.1980	42.0000	35.0000	9.1148	2.6825	0.8906	0.0360	0.8819
**b189**	0.0858	35.0000	29.0000	18.1856	3.0633	0.9475	0.0293	0.9450
**MPGA50**	0.2530	21.0000	17.0000	6.9911	2.2206	0.8573	0.0201	0.8428
**b249**	0.1000	45.0000	33.0000	17.1987	3.0247	0.9426	0.0680	0.9396
**MPGA31**	0.4538	25.0000	16.0000	3.9383	1.8537	0.7497	0.0402	0.7301
**MPGD13**	0.5100	6.0000	6.0000	2.7102	1.1328	0.6310	0.0000	0.5684
**MPGD32**	0.5442	14.0000	8.0000	2.8204	1.3821	0.6509	0.0402	0.6179
**p89**	0.1280	40.0000	28.0000	14.3135	2.8914	0.9305	0.0600	0.9263
**p78**	0.1774	27.0000	19.0000	9.6484	2.4748	0.8952	0.0444	0.8863
**MPGD44**	0.5665	12.0000	8.0000	2.6732	1.3366	0.6279	0.0282	0.5937
**si017**	0.2533	31.0000	25.0000	9.1202	2.5863	0.8904	0.0262	0.8827
**si119**	0.3557	15.0000	11.0000	5.0485	1.8976	0.8019	0.0163	0.7801
**si132**	0.2860	19.0000	14.0000	5.4134	1.917	0.8152	0.0240	0.7914
**si227**	0.1928	23.0000	16.0000	7.5212	2.2133	0.8670	0.0321	0.8529
**b250**	0.0685	46.0000	41.0000	23.8019	3.3637	0.9580	0.0242	0.9563
