# Supplementary material for: Genetic Diversity and Population Structure of Chinese Foxtail Millet [Setaria italica (L.) Beauv.] Landraces
Source: G3 (Bethesda). 2012 Jul 1;2(7):769–77. doi: 10.1534/g3.112.002907 (PMC3385983; doi:10.1534/g3.112.002907)
Supplement: Supporting Information [file supp_2.7.769_TableS2.pdf]

Table S2 New SSR markers used in this investigation

Serial no.	Name of SSR marker	Primer sequence	Repeat type	Chromosoe located	Tm (℃)	Expected size (bp)
1	Si017	F：CCCAGTCACGACGTTGCCGTCGTGCTAGGATACGTT<br>R：AAGGTTGGTAGTTGCCCTTG	(CA)27	5	60	298
2	Si227	F：CCCAGTCACGACGTTGGCGAGCTATACCTCTCACCG<br>R：TGATGGAGCAGCAAGAAATG	(GA)10	8	60	183
3	Si119	F：GCTGAGAAAGTTTGTTGGGC<br>R：GAATTCGAACCGAGCACATT	(AG)18	9	60	293
4	Si132	F：CCCAGTCACGACGTTGCAAATTCTCACCAATGCCCT<br>R：GTTCAGTGGTTGCAAAGGGT	(CA)16	9	60	375
5	B1 (AGDZ23-35)	F: GTTGTCCCTTTCCTCTGTGCTTT<br>R: ATCTCCCGGCGCTTGCTT	(AG)35		53	258
6	X4	F:GTGATGCCACGACACCATAG<br>R: CCTGTTCTTCATCACTGCGA	Dinucleotide		58	313
7	X298	F: CAGAGAGGCATAACGCATGA<br>R: TAGACAACCGATGCAAGCAG	Dinucleotide	9	58	339
